# Functional Properties of Human-Derived Mesenchymal Stem Cell Spheroids: A Meta-Analysis and Systematic Review

**DOI:** 10.1155/2021/8825332

**Published:** 2021-03-31

**Authors:** Sarah Ezquerra, Amparo Zuleta, Rodrigo Arancibia, José Estay, Francisco Aulestia, Flavio Carrion

**Affiliations:** ^1^Cellus Medicina Regenerativa S.A, Complejo Boulevard Kennedy, Av. Presidente Kennedy, 5741 Santiago, Chile; ^2^Cellus Biomédica, Parque Tecnológico de León, C/ Julia Morros s/n, 24009, León, Spain; ^3^Programa de Inmunología Traslacional, Facultad de Medicina, Clínica Alemana Universidad del Desarrollo, Santiago 8320000, Chile

## Abstract

Mesenchymal stem cells (MSC) are adult multi-potent cells that can be isolated from many types of tissues including adipose tissue, bone marrow, and umbilical cord. They show great potential for cell therapy-based treatments, which is why they are being used in numerous clinical trials for a wide range of diseases. However, the success of placebo-controlled clinical trials has been limited, so new ways of improving the therapeutic effects of MSC are being developed, such as their assembly in a 3D conformation. In this meta-analysis, we review aggregate formation, in vitro functional properties and in vivo therapeutic potential displayed by adipose tissue, bone marrow, and umbilical cord-derived MSC, assembled as spheroids. The databases PubMed and SciELO were used to find eligible articles, using free-words and MeSH terms related to the subject, finding 28 published articles meeting all inclusion and exclusion criteria. Of the articles selected 15 corresponded to studies using MSC derived from bone marrow, 10 from adipose tissue and 3 from umbilical cord blood or tissue. The MSC spheroids properties analyzed that displayed enhancement in comparison with monolayer 2D culture, are stemness, angiogenesis, differentiation potential, cytokine secretion, paracrine and immunomodulatory effects. Overall studies reveal that the application of MSC spheroids in vivo enhanced therapeutic effects. For instance, research exhibited reduced inflammation, faster wound healing, and closure, functional recovery and tissue repair due to immunomodulatory effects, better MSC engraftment in damaged tissue, higher MSC survival and less apoptosis at the injury. Still, further research and clinical studies with controlled and consistent results are needed to see the real therapeutic efficacy of MSC spheroids.

## 1. Introduction

Mesenchymal stem cells (MSC) are plastic adherent, fibroblast-like, non-hematopoietic progenitor cells isolated from a variety of adult tissues. MSC have self-renewal capabilities, and they can differentiate into several tissue-specific lineages including osteoblasts, chondrocytes, adipocytes, hepatocytes and cardiomyocytes among others [1, 2]. MSC can be harvested from several tissues, including bone-marrow (BM-MSC), adipose tissue (AT-MSC), umbilical cord (UC-MSC), Wharton's jelly (WJ-MSC), gingiva (G-MSC) and cartilage tissue (C-MSC), among others [3, 4]. The International Society for Cellular Therapy (ISCT) establishes three key features to identify MSC: First, mesenchymal stem cells must display adherence to plastic when cultured under standard conditions. Second, MSC population must express CD105, CD73 and CD90 (≥95%) and not express CD45, CD34, HLA-DR, CD14 or CD11b, CD79a or CD19 (≤2%). Third, MSC cultured in vitro must show osteogenic, adipogenic and chondrogenic differentiation [5]. Besides their ability for multipotent differentiation, MSC are mainly considered as immune evasive cells and they secrete many key trophic factors contributing to tissue repair and regeneration [6–8]. Therefore, MSC appear to be an appealing alternative of treatment in regenerative medicine because it is possible to administrate allogenic populations of these cells or more differentiated lineages [9]. Based on MSC capacity to differentiate and mature into specific phenotypes, their immunomodulatory properties, and their distinct migratory and potent trophic effects during tissue regeneration, the potential for clinical applications is remarkable [10, 11].

At the time of writing this review, there are currently 874 clinical studies reported using MSC to treat different diseases (www.clinicaltrials.gov). However, the beneficial effects of MSC based therapies in small clinical studies are often not substantiated by large randomized double blind, placebo-controlled clinical trials [12, 13]. Several clinical studies, unable to meet the clinical goal, suggest that after prolonged ex vivo expansion of BM-MSC, their immune-suppressive properties change and display deficient survival rate post-transplantation [14, 15]. This implies that it is necessary to comprehend the regenerative and immunomodulatory mechanisms by which MSC exert their action. New ways of enhancing the functional properties of MSC are also needed, which until now have only been cultivated in monolayer 2D culture for clinical applications. Although a simple procedure, some in vivo essential qualities and traits are compromised or lost.

Therefore, 3D cell culture emerges as a new therapeutic alternative, offering better preservation of those features, as allowing a better mimicking of in vivo conditions, particularly cells self-assembling, as observed during embryonic development, thus increasing cells interactions [16, 17]. To promote this interaction, several spheroid formation techniques have been developed. Contrary to monolayer culture, three-dimensional culture of MSC as spheroids causes considerable changes in the gene expression pattern [18, 19]. Diverse studies propose that the functionality of stem cells can be improved and unsuitable migration of cells, after injection in the target tissue, can be avoided, by aggregate formation [20–22]. However, the exact mechanism involved in 3D conformation is still unknown, although several signalling pathways have been proposed [23–28]. For example, it has been described that decreased expression of transcriptional co-activators yes-associated protein/transcriptional co-activator with PDZ-binding motif (YAP/TAZ) in MSC cultured in 3D conformation, was associated with a loss of the actin cytoskeleton [27, 28]. In other study, Zhang et al., showed an increase in the expression of hypoxia-inducible factor (HIF)-1 and -2*α* in MSC spheroids which was related to increased resistance to apoptosis triggered by oxidative stress [23]. This meta-analysis was conducted to review spheroid formation, in vitro functional properties and in vivo therapeutic potential displayed by adipose tissue, bone marrow, and umbilical cord-derived MSC, assembled as spheroids.

## 2. Materials and Methods

### 2.1. Search Strategy

The following databases, PubMed and SciELO, were searched for eligible published articles until May 2019, using specific free-words and MeSH terms. Different combination of the following terms were used: type of cell: mesenchymal, stromal, stem cell, pluripotent, multipotent; cell organization: 3D, spheroid, cluster, organoid, aggregates; application: cell therapy, tissue regeneration, treatment, therapy, functional recovery; pathologies: skeletal muscle, muscle cartilage, tendon or joint pathologies; osteoarthritis, chronic injuries, immune diseases, neurodegenerative diseases, neurological pathology, bone pathology; culture conditions: hypoxia, low oxygen, xeno-free, serum-free, animal-free; properties: secretome, exosome, vesicles, immunoregulatory. Of the results obtained only articles published in English were included and, articles related to cancer and published earlier than 2008 were excluded. Other potential articles were identified from references within the selected articles or reviews related to the topic.

### 2.2. Selection Criteria

Inclusion criteria are listed as: a) Human MSC; b) source of MSC either bone marrow, adipose tissue or umbilical cord; c) studies focused on cell therapy and regenerative medicine. Exclusion criteria are listed as a) article does not meet inclusion criteria; b) reviews and case reports; c) work focused on application and/or use of MSC spheroids in bioengineering.

### 2.3. Data Extraction

The data extracted from included articles consisted of: authors, year, country, title, MSC tissue source, cell aggregation protocol, culture conditions, spheroid measurements, functional properties in vitro, altered markers for said properties, therapeutic effects in vivo and study model used.

### 2.4. Limitations

Only articles published in English were included, which may leave out other eligible publications that were reported in other languages. Therefore, the results should be interpreted cautiously due to the limited data.

## 3. Results and Discussion

### 3.1. Search Results

**Figure 1:**
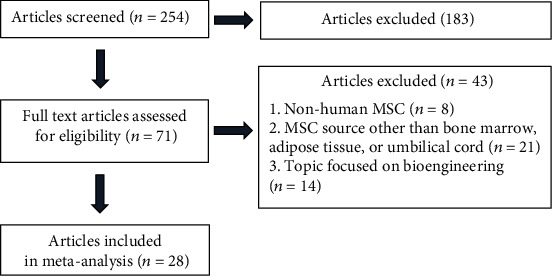
Meta-Analysis Study Selection.

After a screening of 254 articles identified by searching in PubMed (n =131) and SciELO (n =123) associated with MSC in 3D conformation, only 71 articles were assessed for eligibility according to the search strategy described in materials & methods. Of these 71 articles, only 28 articles were incorporated in this meta-analysis according to the inclusion criteria described in materials & methods (human MSC, MSC harvested from bone marrow, adipose tissue or umbilical cord, and studies focused on cell therapy and regenerative medicine (Figure 1).

The list of the 28 articles chosen and their basic description (author(s), year, country, source of MSC, properties and parameters evaluated, and ref number) is shown in Table 1.

It can be noted that the earliest work included in this review is from May 2010 [29] and the latest being published in April 2019 [30]. Most articles are published in 2017 (n =5), succeeded by 2014, 2016 and 2018, each with 4 articles. The most articles collected are from work conducted in the USA with a total of 13 published articles comprising a 46.4% of all articles included, followed by South Korea with a total of 5 (17.9%) and China with 3 (10.7%) (Table 1). Of the 28 articles selected 15 corresponded to studies using MSC derived from bone marrow, 10 from adipose tissue and 3 from umbilical cord blood or tissue.

In all, the most evaluated characteristics of MSC spheroids are stemness, angiogenesis, differentiation, cytokine secretion, paracrine effect, metabolic function, and immunomodulatory effects (Table 1).

### 3.2. Cell Aggregation Protocol

**Figure 2:**
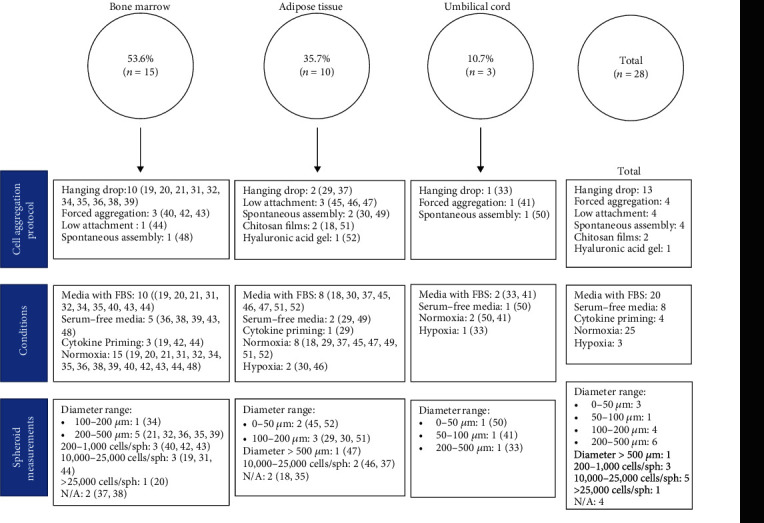
Cell aggregation protocols, culture conditions, and spheroid diameter for selected articles by source of MSC. Article reference number in parenthesis.

There are several methods and techniques to form MSC spheroids, here we have found that the most used procedure is the hanging drop method (n = 13) [19–21, 29, 31–39], followed by forced-aggregation (n = 4) [40–43], low attachment(n = 4) [44–47], spontaneous assembly (n = 4) [30, 48–50], then chitosan films (n = 2) [18, 51], and finally hyaluronic acid gel (n = 1) [52] (Figure 2). The hanging drop technique consists of plating the MSC suspension in droplets of determined volume on the lid of a culture dish. The lid is turned over in a swift and careful move and placed on top of the culture plate which is filled with a solution to avoid drop evaporation. The spheroid is formed in the apex of the drop. This method can yield spheroids of controlled sizes, determined by the number of cells in each drop associated with the concentration of the cell suspension and volume of droplet, and there is no need for specialized and expensive equipment [32, 38]. Even though this technique shows many advantages it still poses a problem for large scale production of MSC spheroids for therapeutic applications [53, 54]. Other of the techniques used for the formation of MSC spheroids is the forced aggregation method which consists in applied centrifugal forces to induce MSC in vitro aggregation using micro-well plates in presence or not of biomaterials [40, 42, 43]. On the other hand, it has been showed that immunomodulatory activity of MSC does not seem to be spontaneous but requires MSC to be ‘licensed' by inflammatory microenvironment to exert their effects [11, 55]. In this line, Krampera and Ren demonstrated that MSC-mediated immunosuppression requires preliminary activation of the MSC by immune cells through the secretion of the pro-inflammatory cytokine IFN-g, alone or together with TNF-a, IL-1a or IL-1b [56, 57]. In this review, 4 of 28 articles selected, use cytokine priming to improve the functional properties of MSC (Figure 2) [29, 42–44].

### 3.3. Culture Conditions

For MSC spheroids to be eligible for clinical applications they need to be xeno-free (not containing any animal-derived components). Serum containing media can carry unexpected agents risking viral or mycoplasma contamination [48]. Then, spheroid formation in media without FBS (fetal bovine serum) is critical. In this analysis, we identified that 8 of the 28 articles included, used serum-free media in their cell aggregation protocols (Figure 2). The importance of FBS is highly noted for cell aggregation during spheroid formation, correlated with spheroids exhibiting faster assembly and more defined edges. Some of the strategies used to substitute FBS include using chemically defined media, in which all components are known, and composition can be controlled or supplementing media with patient-derived serum or human serum albumin [39].

Also controlling the oxygen level during spheroid assembly can have beneficial effects. Hypoxia as a priming method of MSC aggregates was used in three articles [30, 33, 46] (Figure 2). Some research has pointed out that hypoxic conditions during cell aggregation can improve MSC properties. For example, the enhancement of the paracrine effect due to higher levels of IL-6, IL-8, and MCP-1 [46]. From a physiological point of view, MSC cultured under hypoxic conditions can better prepare cells for an ischemic environment typical of damaged tissue.

### 3.4. Spheroid Diameter

Surprisingly, there does not seem to be homogeneity of spheroid size, 6 articles show a similar diameter range of 200-500 *μ*m, 4 articles a diameter range between 100-200 *μ*m, 3 a diameter between 0-50 *μ*m, 1 has spheroids with diameter between 50-100 *μ*m and finally 1 article with spheroid diameter> 500 *μ*m (Figure 2). Another measurement used to describe spheroid size is the number of cells within spheroids, and 5 articles displayed spheroids with 10,000-25,000 cells/spheroids, 3 with 200-1,000 cells/spheroids and 1 with cell concentration greater than >25,000 cells/spheroids (Figure 2). This is the initial number of cells at the beginning of the aggregation process. Some articles (n = 4) made no mention of MSC spheroid diameter or size (Figure 2). Spheroid size is important because the diameter can determine nutrient and oxygen availability, as well as mechanical forces created by the cell-to-cell contacts between MSC, modulating gene expression [58].

### 3.5. In Vitro Properties of MSC Spheroids

Different approaches used to assess the functional properties of MSC can be identified from the selected works. These traits can be studied by variations in gene expression, protein secretion, surface marker expression, culture, and differential staining. Stemness is usually evaluated by the expression of transcription factors Nanog, Oct-3/4, Sox-2, Klf4, c-Myc, and STAT3, but also the expression of surface markers like CD105, CD90, CD73, and CD34 among others [16, 18, 50]. The effect of MSC over angiogenesis, the development of new vessels, is assessed mainly by the expression of the factors VEGF and HGF, among others (Table 2). Differentiation potential to several types of tissue is also studied as a characteristic trait of MSC, generally by different staining techniques like Alizarin Red, Alcian Blue, and Oil Red O staining, to determine osteogenic, chondrogenic and adipogenic differentiation, respectively. Cytokines and soluble factors secreted by MSC has been attributed to great therapeutic effects. Among the cytokines analyzed are growth factors, chemokines, interleukins, and more (Tables 1 and 2). There is also evidence of the paracrine anti-cancer, anti-apoptotic and resistance to oxidative stress of MSC (Tables 1 and 2). There seems to be a great focus on immunomodulatory effects of MSC, so the expression of TSG-6, PGE2, LIF, IDO, STC1, IL-6, IL-8 and more, is normally evaluated, as well as the capacity of MSC to decrease levels of TNF- *α* (Table 2).

### 3.6. In Vivo Therapeutic Effects of MSC Spheroids

Of the total pool of articles reviewed, only 13 assessed the therapeutic effects of MSC spheroids in vivo (Table 3), 2 articles used BM-MSC, 8 articles used AT-MSC and 3 articles used UC-MSC. Several study models are used to investigate in vivo effects, such as wound healing or pro-inflammatory disease models, but mostly ischemia animal models are used (Table 3). In vivo studies include spheroid transplantation or application of conditioned media derived from spheroid culture, into the target tissue. Overall studies reveal that the application of MSC spheroids in vivo has enhanced therapeutic effects compared to monolayer 2D culture. Studies showed better outcomes for MSC in 3D conformation including reduced inflammation, faster wound healing and closure, functional recovery and tissue repair due to immunomodulatory effects, better MSC engraftment in damaged tissue, higher MSC survival and less apoptosis at injury (Table 3).

### 3.7. Limitations of This Study

There are some limitations to this study. First, there is a degree of heterogeneity in the sources of cells and techniques used to produce the MSC spheroids. Second, there are many publications excluded due to technical factors, but still holding conclusions that may be relevant to this research. Finally, there is still a lack of clinical research to test MSC real benefits on a large scale randomized controlled trials.

## 4. Conclusion

Based on the literature reviewed, we can conclude that the most used source of MSC to produce spheroids is bone marrow followed by adipose tissue and umbilical cord. Although MSC can be derived from many adult tissues such as bone marrow, adipose tissue, umbilical cord, and placenta, these sources present some limitations due to differences between donors, extensive in vitro cell culture expansion and clinical trials with inconsistent results [14, 59]. In this line, MSC derived from human Induced pluripotent stem cells (iPSC) has emerged in the last decade as an excellent therapeutic strategy to overcome the limitations of MSC source, inter-donor *variability*, senescence and culture [60–62]. Human-iPSC-derived MSC possess higher proliferative potential and telomerase activity as well as immunomodulatory and angiogenic properties which has been demonstrated in different preclinical models of disease and clinical trials [61, 63–66]. Interestingly, Ding et al., demonstrated that murine-iPSC-derived MSC cultured in an encapsulated 3D spheroid format display stronger immunomodulation in a murine heart transplantation model [67].

The cell aggregation protocol most used was the hanging drop technique followed by forced-aggregation, low attachment, spontaneous assembly, then chitosan films and hyaluronic acid gel.

The evidence here shows MSC traits are enhanced by 3D culture. Results from the 28 articles selected suggest that MSC display better stemness, angiogenesis, differentiation potential, cytokine secretion, paracrine and immunomodulatory effects when presented as spheroids. At the time of this review, no human trials using MSC spheroids were found at the National Institutes of Health (NIH) website.

In this context, genetically engineering MSC or three-dimensional culture, express and secrete important paracrine and immunomodulatory factors such as IDO, PGE2 and TSG-6 suggesting that this might increase the in vivo therapeutic effect of MSC [23, 31, 42, 43].

In comparison with monolayer culture, MSC in 3D conformation presents as an attractive alternative for therapeutic applications of MSC. However, more studies are needed to evaluate the real therapeutic efficacy of MSC spheroids as well as mechanisms and pathways involved.

## Figures and Tables

**Table 1:** Description of published articles included in analysis. For each published article the author(s), year, country, tissue source of MSC, properties and parameters evaluated, and ref number is shown.

Author(s)	Year	Country	Source of MSC	Properties and parameters evaluated for MSC spheroid	Reference
Alimperti *et al.*	2014	USA	Bone marrow	Proliferation and differentiation potential (osteogenic, Adipogenic and Chrondogenic), surface markers expression, serum-free culture.	[48]
Amos et al.	2010	USA	Adipose tissue	Gene expression and proteins levels determination, in-vivo therapeutic potential (diabetic wound model).	[29]
Bartosh et al.	2010	USA	Bone marrow	Antiinflammatory and antitumorigenic molecules expression, in-vitro and in-vivo therapeutic potential (peritonitis model).	[21]
Bartosh *et al.*	2013	USA	Bone marrow	Immunomodulatory factors secretion, IL1 signaling, in-vitro anti-inflammatory effects (macrophage immune assay), in-vivo MSC sphere-like formation.	[31]
Bartosh *et al.*	2014	USA	Bone marrow	MSC spheroid characterization, immunomodulatory factors detection, in-vitro macrophage immune assay.	[32]
Bhang *et al.*	2012	South Korea	Umbilical cord blood	Angiogenic factors secretion, apoptotic/antiapoptotic gene expression, in-vivo therapeutic potential (hindlimb ischemia model).	[33]
Cesarz et al.	2016	USA	Bone marrow	BMP2, IL1 and elasticity-associated signaling, growth factor, cytokine and wound healing-related gene expression.	[19]
Cheng et al.	2012	Taiwan	Adipose tissue	Stemness, proliferation and differentiation potential, in-vivo engraftment (nude mice model).	[51]
Cheng et al.	2013	Taiwan	Adipose tissue	Stemness, angiogenesis, and chemotaxis potential, adipogenic and osteogenic differentiation potential, in-vivo therapeutic potential (wound healing model).	[18]
Cho et al.	2017	South Korea	Adipose tissue	Apoptotic markers and growth factors expression, in-vivo therapeutic potential (elastase-induced emphysema model).	[49]
Costa et al.	2017	Portugal	Bone marrow	Oxidative stress, angiogenic, chemotactic and wound healing potential, immunomodulatory factors.	[40]
Coyle et al.	2019	USA	Adipose tissue	Glucose, ATP and lactate evaluation, metabolic substrates analysis, mathematical modeling.	[30]
Jiang et al.	2017	China	Bone marrow	Cell preservation and survival analysis, transcriptomic analysis, immunomodulatory activity, in vivo therapeutic potential (colitis model).	[34]
Kim et al.	2018	South Korea	Bone marrow	Exosome production, MSC spheroid size, cell density and morphology evaluation.	[35]
Lawrence *et al.*	2019	USA	Bone marrow	Osteogenic differentiation potential, differentiation markers expression.	[36]
Lee et al.	2016	South Korea	Adipose tissue	Hypoxia-induced angiogenic cytokines and extracellular matrix components expression, in-vivo proliferation potential (hindlimb ischemia model).	[45]
Li *et al.*	2015	China	Umbilical cord tissue	Stemness, proliferation and differentiation potential, metabolic analysis, in-vivo therapeutic potential (CCl4-induced acute liver failure model).	[50]
Mineda et al.,	2015	Japan	Adipose tissue	Stemness, angiogenic and antiinflammatory gene expression markers, in-vivo therapeutic potential (ischemia-reperfusion injury, SCID mice).	[52]
Miranda *et al.*	2019	Portugal	Umbilical cord tissue	Secretome production, cytokine/chemokine secretion, migration and differentiation potential, in-vivo therapeutic potential (adjuvant-induced arthritis model).	[41]
Oberringer *et al.*	2018	Germany	Adipose tissue	Cytokine gene expression and protein levels, adipogenic potential, tissue healing-associated angiogenesis potential.	[46]
Park *et al.*	2017	South Korea	Adipose tissue	Photobiomodulation, Angiogenic potential, endothelial and smooth muscle cell differentiation potential, in-vivo therapeutic potential (skin flap model).	[47]
Redondo-Castro *et al.*	2018	UK	Bone marrow	Interleukin-1 priming, trophic factors and cytokine secretion, Angiogenic, regenerative and immunomodulatory potential.	[44]
Xu *et al.*	2016	China	Adipose tissue	Angiogenic, anti-apoptotic, anti-oxidative factors and cytokine secretion, in-vivo therapeutic potential (ischemia-reperfusion kidney injury model).	[37]
Ylostalo *et al.*	2012	USA	Bone marrow	In-vitro immunomodulatory potential (macrophage immune assay), conditioned medium production, anti and pro-inflammatory cytokine secretion.	[20]
Ylostalo *et al.*	2014	USA	Bone marrow	Immunomodulatory potential, IL1 signaling molecules expression, Cancer cell growth effect.	[38]
Ylostalo *et al.*	2017	USA	Bone marrow	Secretome production, antiinflammatory and anti-cancer factors secretion, in-vitro and in-vivo immunomodulatory potential (acute systemic inflammation, LPS).	[39]
Zimmermann *et al.*	2014	USA	Bone marrow	IFN-g and TNF-a priming, immunomodulatory paracrine factors secretion, in-vitro immunomodulatory potential (macrophage immune assay).	[42]
Zimmermann *et al.*	2017	USA	Bone marrow	IFN-g priming, IFN-g microparticle delivery, immune factors secretion, in-vitro immunomodulatory potential (T-cell activation and macrophage immune assays).	[43]

**Table 2:** *In vitro* functional properties of MSC spheroids.

Source of MSC	Functional properties	
Bone marrow (ref)	*In-vitro*	Main findings
48	Serum-free spheroids maintained MSC phenotype. High differentiation potential (3D versus 2D culture conditions).	Positive for surface markers (CD105, CD90, CD73, and CD34). Higher osteogenic, chrondogenic and adipogenic differentiation capacity.
21	High secretion of anti-inflammatory and antitumorogenic factors (3D versus 2D). MSC derived from spheroids retains the properties of 2D MSC. High anti-inflammatory effect in a mouse macrophage immune assay (3D versus 2D).	Higher secretion of TSG-6, STC1, LIF and IL-24, TRAIL. Similar proliferation, immunophenotype and differentiation potential. Higher inhibition of TNF-a secretion by macrophages.
31	MSC form sphere-like structures after i.p. injection of adherent MSC in a mouse. High gene expression of anti-inflammatory factors and IL1 as well as notch signaling molecules. Activation of caspase-dependent IL1 signaling (3D versus 2D). High anti-inflammatory effect after IL1 signaling activation.	High gene expression of COX2, TSG6, and STC1. Up-regulation of TSG-6, COX 2, STC1 and IL1 and notch related molecules. Higher secretion of IL1a, IL1b. Higher secretion of PGE2 and immunomodulatory effect on LPS stimulated macrophages.
32	Protocol for preparation of MSC spheroid.	Efficient MSC spheroids formation using hanging-drop cultures.
19	High gene expression and protein levels of immune, angiogenic and growth factors (3D versus 2D). Decreased expression of immunomodulatory factors after BMP2 treatment. High gene expression of immunomodulatory and growth factors after IL1B treatment.	Higher expression of IL1B, IL8, PTGS2/COX2, TNFAIP6, SOD2, CXCL1, CXCL2, CCL2 and CCL7, BMP2, BMP6. Lower expression of IL1B, IL8, PTGS2/COX2, TNFAIP6, SOD2, CXCL1, CXCL2, CCL2 and CCL7. Higher expression of BMP2, IL1B, IL8, LIF, PTGS2/COX2, TNFAIP6 and SOD2.
40	Increased chemotactic potential induced by conditioned medium (3D, alginate-encapsulated 3D versus 2D). Enhanced immunomodulatory potential. Enhanced angiogenic potential (alginate-encapsulated 3D versus 2D).	Higher migration of fibroblasts in the presence of alginate-encapsulated spheroids. Higher expression of TSG-6 in encapsulated and non-encapsulated spheroids. Higher proangiogenic potential.
34	Enhanced survival under ambient conditions (hESC derived-MSC, 3D versus 2D). Low cell metabolism and proliferation in ambient conditions-recovered MSC. Ambient conditions-recovered MSC retains the properties of 2D MSC.	Higher MSC survival. Lower cell metabolism and proliferation correlates to the enhanced survival. Similar gene expression, immunophenotype, differentiation and immunosuppressive potential.
35	Enhanced secretome secretion (3D versus 2D).	Higher efficiency in exosome production in larger spheroids.
36	High cartilaginous/calcium deposition. Enhanced osteoblast differentiation (3D versus 2D).	Presence of fibrous and mineralized extra-cellular matrix, micro-calcification deposits. Higher osteogenic differentiation capacity.
44	High cytokine secretion after priming with proinflammatory cytokines (3D versus 2D). High cytokine secretion after IL1 priming. High immunomodulatory effect of conditioned medium from spheroids after IL1 priming. Potent immune profile after IL1 priming (3D versus 2D).	Higher secretion of G-CSF, IL-1Ra and VEGF. Higher secretion of IL-6 and G-CSF in 24 hr conditioned media. Decreased TNF-a secretion in LPS treated BV2 microglial cells. Higher expression of MCSF, TNF-b, CC7/MCP3, Gro-a-CXCL1, CCL22, TNF-a, CCL23, IL-6, IL-19, IL-8, MIG/CXCL9 and G-CSF.
20	High immunomodulatory effect (spheroid, spheroid-derived cells and their conditioned medium versus 2D). High immunosuppressive effect of conditioned medium (3D versus 2D). High anti-inflammatory activity.	Effective suppression of TNF-a secretion in LPS-stimulated macrophages. Decreased of TNF-a, CXCL2, IL-6, IL12p40, IL-23 and increased IL-10 and IL1ra secretion. High secretion of PGE2, dependent of COX-2 and mediated by EP4 receptor.
38	Enhanced characteristics in xeno-free medium supplemented with HSA. High anti-inflammatory effect (conditioned medium). High gene expression of immunomodulatory and anti- cancer related molecules (3D versus 2D). High anti-cancer in vitro effect (spheroid-conditioned medium).	High cell viability, cell yield and small cell size. Decreased TNF*α* and increased IL-10 secretion by LPS-stimulated macrophages. High PGE2 and TSG-6 secretion. Higher expression of PGE2, TSG-6, TRAIL, IL-24 and lower levels of TNF-a. reduced prostate cancer cell growth (LNCaP cells).
39	High expression of anti-inflammatory and anti-cancer molecules. High anti-inflammatory and anti-cancer effect (conditioned medium).	Upregulation of PGE2, TSG-6, TRIAL and IL-24. Decreased TNF*α* and increased IL-10 secretion by LPS-stimulated macrophages. Decreased IFN-g production by CD3-stimulated splenocytes. Reduced prostate cancer cell growth (LNCaP cells).
42	High secretion of immunomodulatory paracrine factors (3D versus 2D). High secretion of immunomodulatory factors after TNF-a and/or IFN-g licensing (3D versus 2D and two different cell culture medium). High immunosuppressive effect after TNF-a and/or IFN-g licensing (3D versus 2D).	Higher secretion of PGE2, TGF-*β*1, IL-6 and similar IDO activity. Higher secretion of PGE-2, IL-6 and IDO activity in spheroids cultured in FBS media. Higher suppression of TNF-a secretion by LPS-stimulated macrophages after TNFa/IFN-g priming.
43	High sustained immunomodulatory activity (microparticle delivery of IFN-g within spheroids). High anti-inflammatory effect in a mouse macrophage assay.	High sustained IDO expression and enhanced suppression of T-cell activation and proliferation. Decreased TNF*α* and increased IL-10 secretion by macrophages.
Adipose tissue		
29	High expression of proteins related to angiogenesis, proliferation, migration and ECM deposition (3D versus 2D).	Higher expression of fibronectin, fibrinogen, TIMP1, MMP2, TGF*β*1, FGFb, IGFBP-1, EGF, HGF, VEGF, MMP14, tenascin C, and collagen VI alpha 3.
51	High cell survival and ECM molecules secretion (spheroid formation on chitosan films, 3D versus 2D). Enhanced stemness, proliferation and differentiation potential (3D versus 2D).	Higher viability and laminin and fibronectin secretion. Higher gene expression and proteins levels of Nanog, Sox-2, Oct-4. Higher expansion efficiency, colony-forming activity and osteogenic and adipogenic differentiation as well as transdifferentiation capacity.
18	Enhanced stemness, angiogenic and chemotactic potential (3D versus 2D).	Higher cell growth rate and lower senescence. Higher gene expression and protein levels of Sox2, Oct4, Nanog, HGF, and VEGF. Higher expression of CXCR4, MMP-9 ans MMP-13.
49	Enhanced apoptosis resistance and secretion of growth factors (3D versus 2D).	Higher expression of BCL2, FGF-2, VEGF. Higher BCL-2/Bax ratio. Higher protein levels of VEGF.
30	Spheroid survival potential under varying glucose and oxygen concentrations (mathematical modeling).	High linear correlation between spheroid glucose availability and viability.
45	High angiogenic potential (3D versus 2D). Enhanced resistance to anoikis (3D versus 2D).	Higher expression of VEGF, HGF, SDF-1, HIF-1a, fibronectin and laminin. Higher AKT phosphorylation and lower expression of PARP-1 and cleaved caspase-3.
52	Enhanced stemness, angiogenic and anti-inflammatory potential (spheroids prepared in a HA gel versus 2D) .	Higher gene expression of VEGFA, VEGF B, HGF, PDGFA, PDGFC, IL1RN, IL11 and NANOG, OCT3/4, STAT3 markers. Higher expression SSEA-3 stem cell marker.
46	Enhanced paracrine and regenerative effect.	High gene expression of IL-6, IL-8 and VEGF in response to hypoxia.
47	High Angiogenic and tissue regeneration potential after photobiomodulation irradiation (3D versus 2D).	Higher secretion of FGF, VEGFand HGF. Higher positivity for CD34, CD31 and KDR.
37	High regenerative, anti-apoptotic and anti-oxidative potential (3D, 3D-derived cells versus 2D). Enhanced secretion of cytokines and immunomodulatory factors (3D, 3D-derived cells versus 2D).	Higher secretion of collagen I, fibronectin, laminin. Higher expression levels of catalase, SOD-1, Bcl-2, P-akt al lower expression of cleaved Caspase3. Higher secretion of VEGF, EGF, IGF, bFGF, HGF and TSG-6.
Umbilical cord		
33	Enhanced osteogenic and anti-apoptotic potential. High survival and anti-apoptotic effect under hypoxic conditions (3D versus 2D).	High production of CEGF, FGF2, HGF and Bcl-2 expression. Higher cell viability and Bcl-2 expression.
50	Enhanced stemness, proliferation and differentiation potential (3D versus 2D).	Higher expression of *Klf-4*, C-myc. Higher osteogenic and adipogenic differentiation potential.
41	High anti-inflammatory effect of conditioned medium (3D versus 2D). High motogenic effect of conditioned medium over mouse chondrocytes (3D versus 2D).	Higher levels of anti-inflammatory and trophic factors (IL-10, LIF, PDGF-BB, FGF-2, I-309, SCF, GM-CSF). Higher chondrocyte migration capacity.

**Table 3:** *In vivo* therapeutic effect of MSC spheroids.

Source of MSC	Therapeutic effect	Animal study model
	In vivo main findings	
Bone marrow (ref)		
21	Higher anti-inflammatory effect, 3D versus 2D, (spheroid treatment decreased neutrophil activity, and TNF-a, IL-1b, CXCL2/MIP2 and PGE2 levels.	Murine zymosan-induced peritonitis.
34	Ambient conditions-recovered MSC retains therapeutic effect (3D versus 2D).	DSS and TNBS-induced mouse colitis model.
39	High anti-inflammatory effect (enhanced PGE-2 and IL-10 and decreased TNF-a levels).	LPS-induced systemic inflammation in mice.
Adipose tissue		
29	Enhanced wound healing (faster wound closure, 3D versus 2D), higher production of tenascin C, collagen VI a3, fibronectin, MMP2, MMP14 and HGF.	Diabetic mouse wound model.
51	High therapeutic effect. Higher cellular retention after intramuscular injection into hindlimbs (3D versus 2D).	Nude mice model.
18	Higher regenerative capacity (3D versus 2D).Enhanced cutaneous wound closure, cell engraftment and angiogenesis.	Wound healing nude mice model.
49	Enhanced therapeuti**c** and regenerative effect (3D versus 2D). Greater regeneration of lung tissues and higher FGF2 and HGF expression.	Elastase-induced emphysema mouse model.
45	Higher cell survival and proliferation in ischemic tissue (3D versus 2D).	Murine hindlimb ischemia model.
52	Enhanced therapeutic and regenerative effect (3D versus 2D). Spheroids promoted tissue repair and reduced the final atrophy.	Ischemia-reperfusion injury in SCID mice.
47	Enhanced survival and therapeutic effect (3D versus 2D). Higher survival, angiogenic efficacy and differentiation into epithelial cells. Greater effectiveness in functional recovery of ischemic skin flap.	Murine ischemic skin flap model.
37	Enhanced survival, paracrine secretion and therapeutic effect (3D versus 2D). Higher secretion of VEGF, HGF and TSG-6. Less apoptosis and tissue damage at injury site and higher vascularization.	Ischemia-reperfusion injury in rats kidneys.
Umbilical cord		
33	High regenerative and therapeutic effect (3D versus 2D). Enhanced cell survival, angiogenesis and cell adhesion molecules and growth factors expression (VEGF, FGF2, ICAM, VCAM and NG2). Spheroids transplantation improved limb salvage and attenuated fibrosis.	Mouse hindlimb ischemia model.
50	Enhanced regenerative and therapeutic effect (3D versus 2D). Faster decreased of ALT, AST and total bilirubin. Spheroid treatment increases IFN-g and IL-6 serum levels and reduces TNF-a levels.	CCl4-induced acute liver failure murine model.
41	Higher therapeutic effect of conditioned medium (3Dversus 2D), spheroid-derived conditioned medium attenuated tissue destruction, reduced synovial inflammation and bone erosion.	Adjuvant-induced arthritis in wistar rats.
